# REFINING CARE NETWORK MEASURES FOR DIVERSE DEMENTIA CAREGIVERS USING CROSS-CULTURAL COGNITIVE INTERVIEWING

**DOI:** 10.1093/geroni/igae098.3887

**Published:** 2024-12-31

**Authors:** Melissa Howe, Alan Rodriguez Tiburcio, Kelly Pudelek, Selena Zhong, Nell Compernolle, Lissette Piedra

**Affiliations:** NORC at the University of Chicago, Penn Valley, California, United States; NORC at the University of Chicago, NORC at the University of Chicago, Illinois, United States; NORC at the University of Chicago, Chicago, Illinois, United States; NORC at the University of Chicago, Chicago, Illinois, United States; NORC at the University of Chicago, Chicago, Illinois, United States; University of Illinois Urbana-Champaign, University of Illinois at Urbana-Champaign, Illinois, United States

## Abstract

It is often assumed that American English speakers universally understand validated instruments and survey items used in previous research. However, comprehension can vary widely due to differences in dialect, education background, and other factors, impacting both survey data quality and respondent burden. To address this, we used cross-cultural cognitive interviewing methods to: (1) facilitate the inclusion of caregivers from diverse cultural and linguistic groups and (2) ensure that survey items elicit comparable data across these groups for accurate analysis. We conducted ~60-minute interviews with 20 informal caregivers to test new and existing survey questions designed to characterize the care networks and caregiving experiences of adults who regularly assist a family member or friend living with dementia. We assessed their ease or difficulty when answering survey questions with close-coded response options and probed into their understandings of key terms. Initially, we interviewed ten caregivers purposefully selected to represent diverse ethnoracial and gender identities, education backgrounds, and geographic locations (urban/rural). After analyzing the transcripts to identify and remedy problematic wording, we conducted ten additional cognitive interviews with new respondents, focusing on Latinx caregivers who primarily speak American English. This iterative process informed specific changes, such as refining the description of “caregiver” in a question designed to be maximally inclusive and replacing misunderstood words such as “dominant” and “primary” with more familiar words. Variations in participant comprehension underscore the importance of rigorously pre-testing research materials. Post-survey development, our data and findings will provide valuable context for interpreting survey results.

